# Deciphering consumer perceptions of Kisra prepared from biofortified and non-biofortified sorghum cultivars using the rate-all-that-apply (RATA) method

**DOI:** 10.3389/fnut.2025.1563247

**Published:** 2025-03-10

**Authors:** Tilal Sayed Abdelhalim, Manhal Gobara Hamid, Mazahir Shaheen, Claudia Böhme, Khitma A. Sir Elkhatim, Yousif M. A. Idris, Mohammed Hamad, Faraz Muneer, Mahbubjon Rahmatov, Mohammed Elsafy

**Affiliations:** ^1^Biotechnology and Biosafety Research Center, Agricultural Research Corporation, Shambat, Khartoum, Sudan; ^2^College of Agricultural Studies, Sudan University of Science and Technology, Shambat, Khartoum, Sudan; ^3^Leibniz Institute for Educational Media, Georg Eckert Institute (GEI), Braunschweig, Germany; ^4^Al-Gadaref Research Station, Agricultural Research Corporation, Shambat, Gadaref State, Sudan; ^5^Department of Plant Breeding, Swedish University of Agricultural Sciences (SLU), Alnarp, Sweden

**Keywords:** Sudan, malnutrition, Kisra, market segmentation, sensory acceptance

## Abstract

**Introduction:**

Micronutrient deficiency remains a significant public health challenge in developing countries, including Sudan, particularly in rural areas, where access to a well-balanced diet is limited. This study evaluated consumer sensory acceptance and quality of Kisra, a traditional Sudanese fermented flatbread prepared from a combination of biofortified and non-biofortified sorghum cultivars.

**Methods:**

A panel of 100 assessors aged 18–75 evaluated nine Kisra products using hedonic overall liking and the rate-all-that-apply (RATA) technique.

**Results and discussion:**

Kisra made from the biofortified Dahab cultivar received the highest overall liking scores (~8, “like very much”), whereas blends such as Dahab + Dabar and Dahab + Arfa-gadamek showed moderate sensory acceptance (~7). Hierarchical agglomerative analysis grouped the assessors into three clusters comprising 50%, 26%, and 24% of the panel. Among the demographic factors, education level had the most significant influence on overall liking (51%), followed by consumption frequency (25.5%), and age (23.5%). The RATA model demonstrated a high assessor repeatability (0.994), homogeneity (0.904), and low global error (9.617). Sensory attributes, namely porousness, thickness, and sourness, were key drivers of preferences for Kisra made from Dahab. These findings underscore the effectiveness of RATA in the product and scaling biofortified Kisra to combat malnutrition and cater to diverse consumer preferences.

## 1 Introduction

Micronutrient deficiencies, especially anemia, are a major public health challenge in developing countries such as Sudan, affecting women and children under five (1). According to the World Health Organization (WHO), anemia affects 40% of children under five and 37% of pregnant women worldwide, causing cognitive impairment, weakened immunity, stunted growth, and increased child mortality (https://www.who.int/news-room/fact-sheets/detail/anaemia). A recent meta-analysis and systematic review by Idriss et al. (2) found that 62.7% of Sudanese children had unclassified anemia, 39.1% had iron (Fe) deficiency, and 8.7% suffered from sickle cell anemia, indicating a significantly higher prevalence than global averages. These findings underline the urgent need for proactive and effective interventions to address this pressing public health issue (3, 4).

Socioeconomic factors compounded the challenges of combating anemia and malnutrition in Sudan. Fadol et al. (5) reported that conflict, food insecurity, and systemic issues deeply rooted in historical, political, and economic disparities significantly worsen malnutrition. Poor dietary practices and insufficient intake of micronutrient-rich foods further hinder efforts to combat malnutrition (6). Additionally, global funding cuts have reduced the capacity of United Nations (UN) agencies and international non-governmental organizations (NGOs) to supply fortified food, intensifying the crisis (7). These barriers demonstrate the critical need for sustainable and culturally appropriate solutions that address the nutritional and socioeconomic factors. Holistic interventions such as biofortification and nutrition education can improve dietary diversity and effectively address malnutrition (8). Improving food preparation techniques is also essential for mitigating nutrient deficiencies.

Biofortification, achieved through plant breeding to enhance the nutritional quality of staple crops, such as high iron (Fe) and zinc (Zn) varieties, provides a cost-effective solution for rural populations dependent on subsistence farming (9–11). Hence, biofortified crops enriched with Fe and Zn can address micronutrient deficiencies in populations with limited access to supplements and fortified foods. This approach aligns with UN Sustainable Development Goal 2, which seeks to end hunger and malnutrition by 2030 through sustainable agricultural practices (10). The success of biofortification relies on key factors, such as the development of nutrient-rich cultivars, consumer acceptance, and effective dissemination strategies (12–14). Understanding consumer preferences is crucial for increasing community adoption of biofortified products, such as sorghum-based foods. Identifying the sensory attributes that drive acceptance while improving accessibility and affordability is crucial for scaling biofortified crops in Sudan and other regions with high malnutrition rates.

In 2022, Sudan's National Variety Release Committee approved Dahab (Parbhani Shakti), the first biofortified sorghum cultivar introduced by the International Crops Research Institute for the Semi-Arid Tropics (ICRISAT), to combat micronutrient deficiencies. Dahab is enriched in Fe(up to 45 ppm) and Zn (up to 32 ppm), provides higher protein, and has reduced phytate levels (4.1 mg/100 g), which enhances nutrient bioavailability (15, 16). Despite these advancements, Habeych et al. (17) reported that micronutrient fortification can alter sensory attributes, such as flavor, color, and texture, potentially affecting consumer acceptance.

Practical sensory evaluation is critical to understanding consumer preferences for biofortified foods. Conventional methods, such as quantitative descriptive analysis, are effective for profiling sensory characteristics but are time-consuming, costly, and require extensive training (18). There is growing demand for rapid and resource-efficient sensory evaluation methods to accelerate product development and address the needs of vulnerable communities (19). The rate-all-that-apply (RATA) method has emerged as a valuable tool that enables consumers to identify and evaluate product features, while providing detailed insights into sensory perceptions and preferences (20).

RATA is a flexible and nuanced sensory profiling method that combines the benefits of check-all-that-apply (CATA) with intensity scaling (21). This approach enables semi-trained or untrained consumers to provide meaningful data, making it particularly suitable for studies conducted in resource-limited settings (22). Additionally, RATA efficiently captures multidimensional sensory information, making it an effective method for assessing consumer perceptions of biofortified foods, such as Kisra. Its ability to generate rich sensory data with minimal panelist training makes it an ideal approach for evaluating biofortified kisra and optimizing products for consumer acceptance.

Kisra, a traditional fermented flatbread made from sorghum, is a stable food deeply embedded in Sudanese culinary practices. It is consumed daily and during communal gatherings, particularly in rural areas where malnutrition is the most prevalent (23, 24). The preparation of Kisra involves traditional fermentation, which enhances its nutritional value, improves sensory properties, and increases the bioavailability of essential nutrients, such as vitamins and minerals, which are lacking in malnourished populations (25).

The primary objective of this study was to evaluate the sensory attributes of Kisra, a traditional fermented flatbread made from the biofortified sorghum cultivar Dahab and its blends with other Sudanese cultivars. This study systematically assessed consumer preferences and sensory profiles of biofortified and non-biofortified Kisra products using hedonic overall liking scores and the RATA method. By identifying the key sensory attributes influencing consumer acceptance, this study provides valuable insights to guide the development of biofortified products according to local preferences. It also aims to promote the integration of biofortified foods such as sorghum-based Kisra into Sudanese diets as a sustainable strategy to combat micronutrient deficiencies. This strategy aligns with the global efforts to enhance nutritional security and support the adoption of biofortified crops in resource-limited communities.

## 2 Materials and methods

### 2.1 Participants

This study aimed to investigate the usefulness of the rate-all-at-apply (RATA) method to naïve consumers profile a wide range of Kisra products. For this purpose, this research was conducted with a panel of 100 female assessors aged 18–75 years from villages in the Eastern Galabat locality of Al-Gadareif State, Eastern Sudan, to further elucidate the discrimination ability of the RATA. Participants were selected for regular involvement in Kisra preparation and consumption, ensuring informed and relevant sensory feedback. An interview-assisted questionnaire was used to collect detailed demographic information, including age, education level, and frequency of kisra consumption. These data enabled a thorough analysis of how age, education, and consumption habits influence sensory preferences. The structured interviews enabled accurate data collection and accommodated participants with varying literacy levels.

### 2.2 Ethical approval and participant consent

The research protocol for this study was approved by the National Health Research Ethics Committee of the Federal Ministry of Health in Sudan, confirming full compliance with the national ethical standards. Written informed consent was obtained from all participants after they were thoroughly briefed on the objectives, procedures, and rights. Participants were assured of voluntary participation and were informed that they could withdraw without any consequences. To safeguard privacy and confidentiality, it was emphasized that all responses would be used exclusively for research purposes and reported anonymously. These measures followed international guidelines, including the principles of the Declaration of Helsinki, ensuring the integrity of the study and protection of participant rights.

### 2.3 Sorghum cultivars and flour preparation

Grains from five sorghum cultivars (Dahab, Wad Ahmed, Dabar-Tabat, Korokolo, and Arfagdamek-8) were obtained from the sorghum breeding program at Al-Gadarif Research Station, Agricultural Research Corporation (ARC), Sudan, where they were field-grown during the 2023–2024 season. Dahab is a high-yielding, creamy-seeded, biofortified sorghum cultivar with 45 ppm Fe and 32 ppm Zn in its grain (26). It was developed by the International Crops Research Institute for the Semi-Arid Tropics (ICRISAT) and Vasantrao Naik Marathwada Krishi Vidyapeeth (VNMKV) in Maharashtra, India, through progeny selection from the high-Fe and Zn landrace IS 26962, and was released in India as Parbhani Shakti (ICSR 14001) in 2018 (15). In Sudan, Dahab was officially registered in 2022 to combat micronutrient deficiencies in rural areas. The other cultivars, namely, Wad Ahmed, Arfagadamek, Korokolo (Feterita varieties), and Dabar-Tabat, were selected for their popularity as good for making Kisra and widespread consumption in Al-Gadareif State (23). Daber and Dahab are the type 1 (non-tannin) and sorghum cultivars, respectively. In contrast, Feterita cultivars are categorized as white type II tannin sorghum, recognized for their distinct sensory and processing properties (23). According to Abdelhalim et al. (27), the iron content in Sudanese sorghum cultivars ranges from 11.8 to 19.1 ppm, with an overall average of 12.5 ppm. In contrast, Sudanese-released sorghum cultivars have been found to contain very low Zn levels, below 4.5 ppm (28).

Before Kisra preparation, the grains were subjected to a standardized cleaning process to remove debris, dirt, and husk. Subsequently, the grains were washed, sun-dried, and milled into flour by using a commercial mechanical stone mill.

### 2.4 Kisra preparation

Kisra flatbreads were prepared according to the traditional protocol commonly used by Sudanese households (24). Briefly, a thick fermented dough, locally known as “*Ajin*,” was made by mixing 60% sorghum flour with 40% water in a round earthenware container called *Khumara*. The dough was left to ferment for 12–24 h using traditional lactic acid and yeast fermentation, as described by TINAYSP et al. (29), until it developed a sour taste. Fermented Ajin was then diluted to a thin batter by adding 50 ml water to 100 g of Ajin. To promote the adoption of the newly released biofortified sorghum cultivar Dahab while preserving the traditional taste of Kisra, a 50:50 blend of flour from Dahab and conventional Sudanese sorghum cultivars was used.

**Figure 1:**
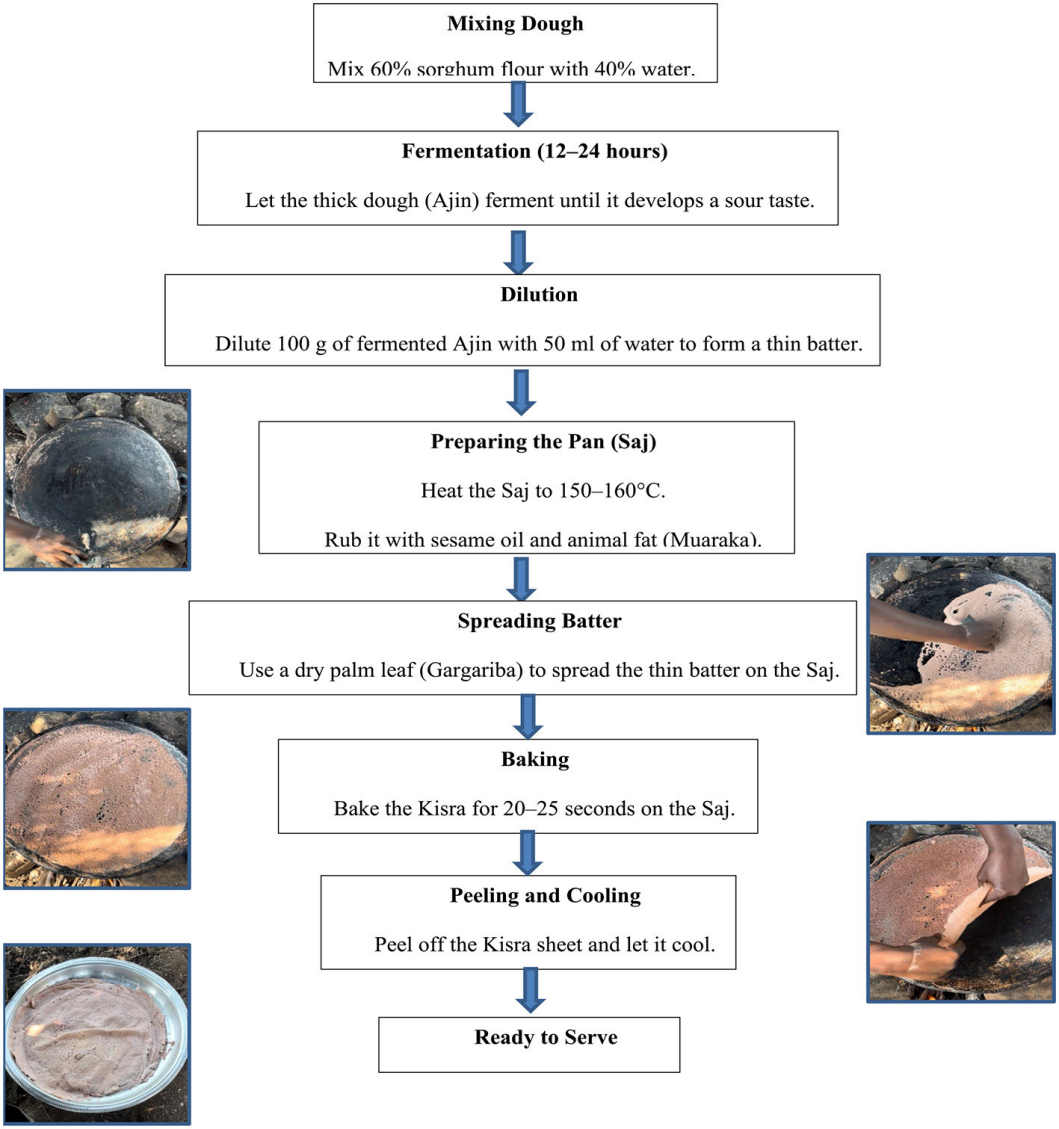
Flowchart depicting Kisra, a fermented flatbread preparation step.

This batter was baked on a flat steel pan (*Saj*) heated to 150–160°C over an open fire fueled by sorghum and charcoal using a unique Sudanese process known as Awoasa. Before spreading the batter, the *Saj* was rubbed with a cloth dampened with sesame oil and animal fat, locally known as Muaraka, to ensure even spreading. The batter was spread with a piece of dry palm leaf (G*argariba*) until a very thin soft sheet of Kisra was formed. After being left on the hot *Saj* for 20–25 s, the sheet was peeled off and considered ready to eat, which was thoroughly followed to maintain consistency and standardization across all samples (Figure 1).

### 2.5 Sensory evaluation

#### 2.5.1 Hedonic test

For the hedonic evaluation, nine Kisra products prepared from five sorghum cultivars, specifically the biofortified Dahab cultivar and its blends with the other four cultivars, were prepared and served on sample plates with codes for each sample. Kisra samples were served on coded plates, with each sample assigned a unique three-digit random code to prevent bias and ensure clarity in identification. Consumer feedback was collected using a nine-point hedonic scale, as described by Peryam and Pilgrim (30), with anchors ranging from 1 (“not at all satisfied”) to 9 (“extremely satisfied”).

The hedonic evaluation was conducted in two sessions, with each panelist independently assessing all the nine samples. The testing environment was carefully controlled, maintaining a room temperature of 23 ± 1°C and relative humidity of 40–50% to ensure optimal sensory conditions. The panelists were seated individually with adequate spacing to prevent interactions or discussions during the evaluation and ensure unbiased assessments. Each panelist was given a 5 min break after evaluating every three samples to minimize sensory fatigue and maintain focus. Additionally, water and unsalted crackers were provided for palate cleansing between the samples to prevent carry-over effects and ensure accurate evaluations.

#### 2.5.2 Rate-all-that-apply (RATA)

An attribute generation session was conducted in Wad Dayief Village, Eastern Galabat locality, Al-Gadareif State, Sudan, through focus group discussions (31) with 25 participants to identify the RATA attributes. Participants were selected based on their regular consumption and kisra preparation. To generate the attributes, each participant evaluated nine Kisra samples prepared from the tested sorghum cultivars, labeled A to I. Subsequently, participants were asked to write five attributes that best described each Kisra sample based on their opinions and emotions. Participants were asked to define sensory attributes through visual examples and hands-on exercises using Kisra samples to illustrate lightness vs. darkness, thickness vs. smoothness, and sweetness vs. sourness to ensure clarity and eliminate redundancy. The most commonly mentioned descriptors across all groups were selected to create the RATA attribute list to ensure relevance and familiarity among consumers. Eleven attributes were generated and cateogrized into three sensory domains: appearance (lightness, darkness, reddishness, and porousness), taste (sweetness, sourness, and bitterness), and texture (lumpiness, thickness, smoothness, and hardness).

After selecting the attributes, the RATA test was conducted on 100 naive Kisra consumers. Assessors were instructed to rate the intensity of all relevant terms used to describe the nine Kisra product samples on a 5-point intensity scale (1 = low-intensity,” 3 = medium-intensity, and 5 = high-intensity). Before sensory evaluation, a 15–20 min induction session was conducted to introduce the participants (assessors) to the RATA method, ethical consent, sensory attributes, and Kisra products. To minimize order bias, the presentation of kisra products in the questionnaire was balanced both within and across participants, following the standard protocol (32). Water was provided to the assessors to rinse their mouths between the Kisra samples to minimize carryover effects and ensure accurate evaluation.

### 2.6 Data analysis

Assessor preferences for the nine Ksira products were evaluated by one-way analysis of variance (ANOVA) using XLSTAT statistical software (version 2024, Addinsoft, New York, NY, USA), with overall liking score as the dependent variable and Kisra products as qualitative variables. Significant differences (*P* < 0.05) between Kisra products were determined using Tukey's *post-hoc* pairwise comparison test. To visualize the overall preference of the nine Kisra products to assessors, an internal preference mapping biplot was employed using principal component analysis (PCA) correlation, where the Kisra products were projected in the space defined by the assessors. Euclidean distances and Ward's Hierarchical Cluster Analysis (HCA) were used to group assessors based on their normalized overall liking scores. An intelligence pivot table in XLSTAT was used to connect demographic information, such as age, level of education, and Kisra consumption patterns, with the overall liking scores.

RATA data were analyzed using the analysis of variance (ANOVA) as described by Meyners et al. (33). PCA was used to obtain a bidimensional representation of the samples and sensory attributes (>70% variance explained). Confidence ellipses (95%) around the samples were constructed based on truncated total bootstrapping (34). The stability of the sample and term configurations was assessed using a bootstrapping approach (35).

## 3 Results

### 3.1 Demographic profile characteristics of the assessors

The study's panel of 100 female assessors displayed diverse demographic compositions regarding age, education level, and Kisra consumption patterns. The assessors' ages ranged from 18 to 75 years, with the majority (26%) aged 36–45 years, followed by 18–25 years (23%) and 46–55 years (21%). Smaller proportions of the panel were aged 26–35 (18%), 56–65 years (8%), and 66–75 years old (4%), showing a broad representation across age groups (Table 1).

**Table 1:** Demographic profile characteristics of assessors (*N* = 100) participating in this study.

**Variables**	**Categories**	**Frequency (*N =* 100)**	**%**
Age	18–25	23	23
	26–35	18	18
	36–45	26	26
	46–55	21	21
	56–65	8	8
	66–75	4	4
Education level	Bachelor	10	10
	High school	39	39
	Illiteracy	12	12
	Primary school	29	29
	Secondary school	10	10
Consumption pattern	Once a day	24	24
	Once a week	2	2
	Thrice a week	18	18
	Twice a day	33	33
	Twice a week	23	23

Assessors' educational levels also varied considerably. The majority (39%) had attended intermediate school, whereas 29% had completed primary school. Among the assessors, 12% were illiterate, and only 10% were secondary school graduates or had a bachelor's degree. These findings show a wide range of educational backgrounds within the panel, representing diverse socio-educational backgrounds typical of rural Sudanese communities (Table 1). Most assessors reported kisra consumption daily or multiple times per week. A significant proportion (33%) consumed Kisra twice a day, followed by 24% who consumed it once a day, and 23% who reported eating it twice a week. An additional 18% consumed Kisra three times a week, while only 2% reported a less frequent consumption pattern once a week (Table 1).

### 3.2 Overall liking of the Kisra product samples

The three-way ANOVA indicated significant differences in the overall liking scores among the nine Kisra product samples. Neither assessors nor sessions significantly varied the overall liking scores, showing consistency in the individual and temporal assessments. Significant interactions were observed between Kisra products and sessions, and between assessors and sessions, suggesting that product preferences varied across evaluation times and were affected by assessor perceptions. However, there was no significant interaction between Kisra products and assessors for overall liking scores, indicating that individual product preferences were relatively consistent across participants (Table 2).

**Table 2:** Overall liking scores for nine Kisra products prepared from five sorghum cultivars as influenced by Kisra products, assessors, sessions, and their three-way interactions.

**Source**	**DF**	**Sum of squares**	**Mean squares**	***F***	**Pr > *F***
Product	8	191.351	23.919	3.952	0.032
Assessors	99	691.739	6.987	0.871	0.754
Sessions	1	33.894	33.894	2.830	0.103
Product^*^Assessors	792	1,556.316	1.965	1.070	0.170
Product^*^Sessions	8	47.391	5.924	3.227	0.001
Assessors^*^Sessions	99	781.162	7.891	4.298	< 0.0001
Error	792	1,454.053	1.836		

Model: Y = P + A + S + P^*^A + P^*^S + A^*^S.

Across all assessors, Kisra made from the biofortified sorghum cultivar Dahab received the highest overall liking score (~8 “like very much”), significantly (*P* < 0.05) outperforming the other Kisra samples (Table 3). Kisra prepared from blends such as Dahab + Arfagadamek and Dahab + Dabar, Kisra prepared solely from Arfagadamek, achieved moderately positive preference scores (around 7, “like moderately”). Compared with Dahab alone, the results of these tests were significantly lower (Table 3). Kisra prepared from Dabar, Korokolo, and Wad Ahmed, as well as blends such as Dahab + Korokolo and Dahab+Wad Ahmed and Wad Ahmed recorded negative overall liking scores. Among these, the Dahab + Wad Ahmed blend had the lowest preference score (Table 3).

**Table 3:** One-way ANOVA for overall liking scores for nine Kisra product samples presented to all assessors and different assessor clusters.

**Product**	**All-assessors**	**Cluster 1**	**Cluster 2**	**Cluster 3**
Arfa-gadamk	7.37^B^	7.79^AB^	6.27^C^	7.69^A^
Dabar	7.14^BC^	7.00^C^	7.71^AB^	6.79^BC^
Dahab	8.02^A^	8.20^A^	7.88^A^	7.77^A^
Dahab + Arfa-gadamk	7.39^B^	7.51^B^	7.19^B^	7.33^AB^
Dahab + Dabar	7.43^B^	7.96^B^	7.17^B^	6.58^AB^
Dahab + Korokolo	7.14^BC^	6.85^C^	7.12^B^	7.77^A^
Dahab + Wadahmed	6.87^C^	6.63^C^	7.60^AB^	6.56^C^
Korokolo	6.99^C^	6.63^C^	7.21^B^	7.50^AB^
Wad Ahmed	6.99^C^	6.72^C^	7.13^B^	7.40^AB^
F	17.37	33.72	9.60	9.281
Pr > F	< 0.0001	< 0.0001	< 0.0001	< 0.0001
*p*-values signification	^***^	^***^	^***^	^***^

Different superscript letters within a column correspond to Kisra products, indicating a significant difference according to Tukey's test. Signification codes: 0 < ^***^ < 0.001.

**Figure 2:**
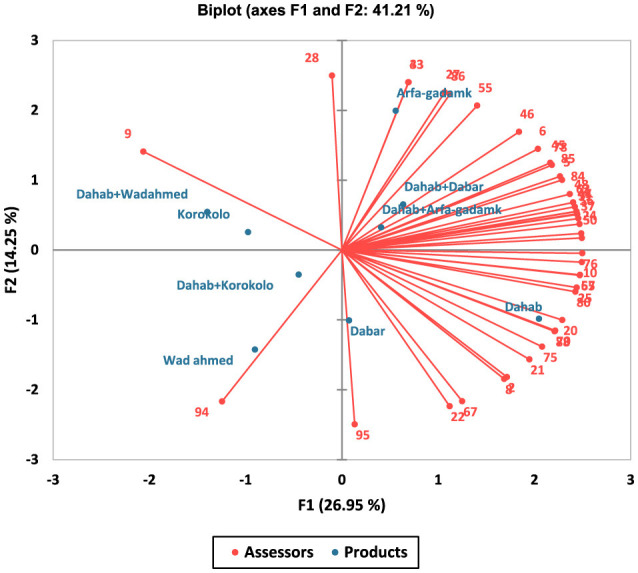
Internal preference mapping depicting the relationship between Kisra products and all assessors, based on overall preferences. The *blue points* indicate the different Kisra samples, whereas the *red vectors* represent the assessors.

The internal preference mapping biplot illustrated the relationship between the assessors' preference feedback and Kisa products (Figure 2). Principal components F1 and F2 accounted for ~41.21% of the total variation, with F1 contributing 26.95%, F2 contributing 26.95%, and F2 contributing 14.25% of the total variation. Kisra, made from biofortified Dahab, was suited to the lower-right quadrant of F1, revealing the highest positive preference among all Kisra products. However, its negative F2 score (−3.717), indicated that it lacked characteristics linked to the positive end of F2. In contrast, Kisra, made by Arfagadamek, was located in the far upper-right quadrant on the F2 axis, showing a clear distinction from Dahab. Blended Kisra products, such as Dahab + Dabar (F1 = 3.304, F2 = 2.458) and Dahab + Arfagadamk (F1 = 2.122, F2 = 1.222), were positioned in the upper-right quadrant, displaying a balanced sensory profile with moderate positive values on both axes.

Wad Ahmed (−4.682, −5.382) and Dahab + Wad Ahmed (−7.261, 2.057) appeared on the negative F1 axis, with Wad Ahmed showing the most negative ratings across both dimensions, indicating distinct sensory characteristics from positively associated Kisra samples. Korokolo (−5.063, 0.970) and Dahab + Korokolo (−2.330, −1.327) occupied the negative F1 quadrant with small F2, suggesting similarity to other left-positioned products while lacking extreme differentiation along F2. Eventually, Dabar (0.386) remained neutral in F1 but had a strongly negative F2 score, indicating unique sensory attributes that set it apart from other samples (Figure 2).

### 3.3 Segmentation of consumers into groups of similar overall liking

**Figure 3:**
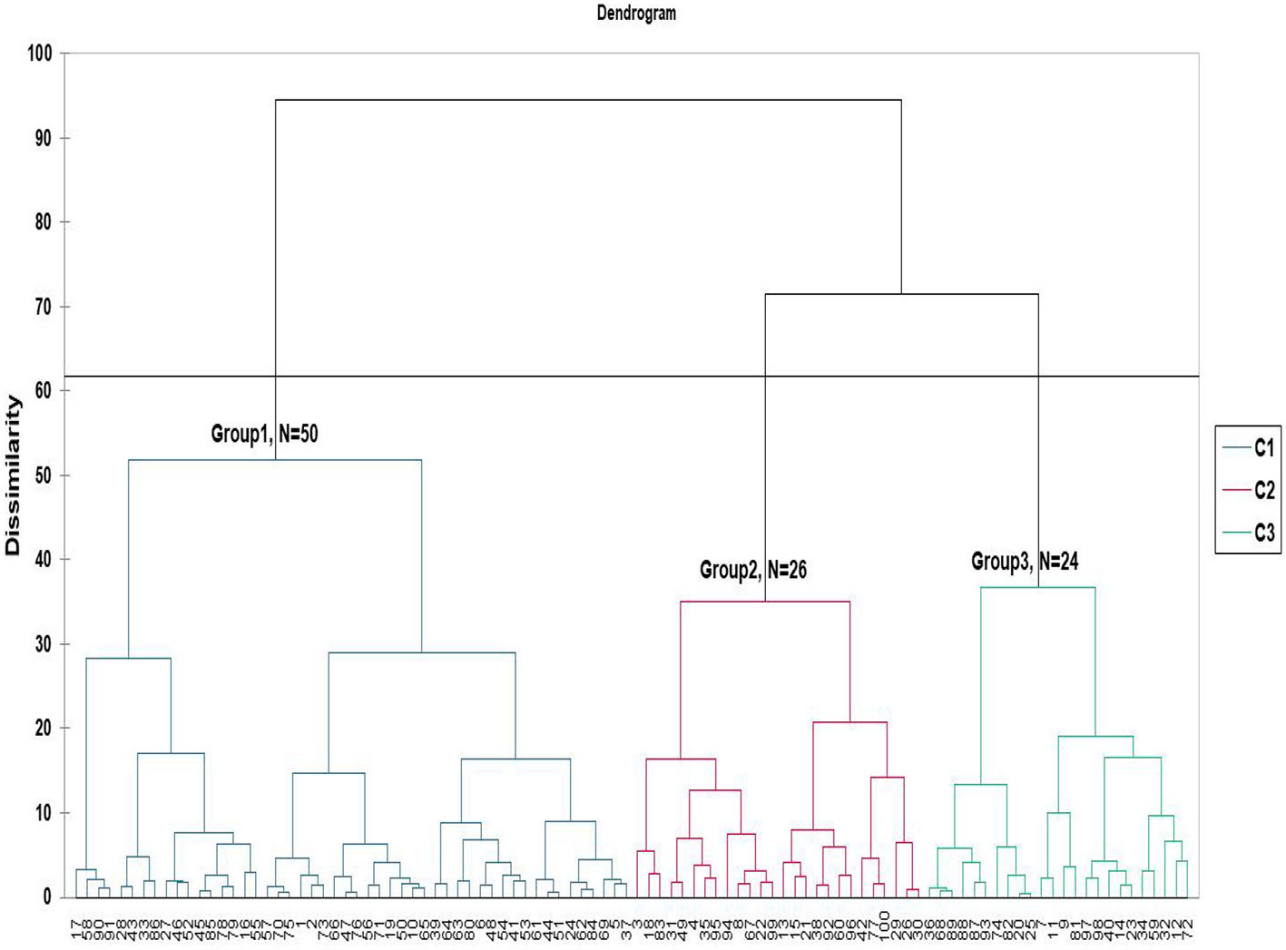
Clustering of the 100 assessors based on their overall liking scores for the nine Kisra products, performed using agglomerative hierarchical clustering analysis. Three groups of assessors were identified: 50%, 26%, and 24% of the total assessors, respectively.

Based on internal mapping preferences (Figure 2), it was evident that there was no clear consensus among assessors, as their preferences were highly dispersed. Therefore, a clustering analysis based on agglomerative hierarchical analysis was applied to segment consumers based on their overall preferences. The dendrogram presented in Figure 3 illustrates the agglomerative hierarchical clustering (AHC) of assessors based on their overall liking preferences for Kisra samples. The y-axis represents dissimilarity, revealing the degree of variation among the assessors' preferences. Clustering analysis grouped the assessors participating in this study into three distinct consumer clusters (Figure 3). Each cluster employed internal preference mapping using PCA to provide insight into variations and consumer preferences.

**Figure 4:**
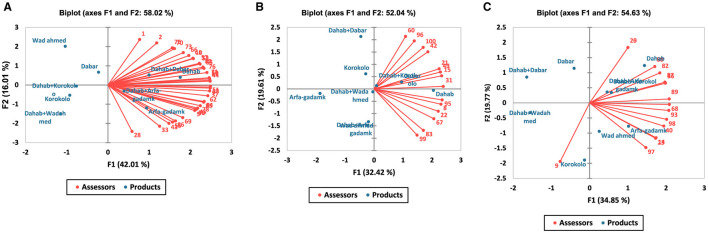
**(A)** Biplots illustrate the internal preference mapping relationships between the Kisra products (*blue points*) and assessors (*red points*) for cluster 1 assessors (50 assessors). **(B)** Shows the internal preference mapping relationships between Kisra products (*blue points*) and assessors (*red points*) for Cluster 2 (26 assessors). **(C)** Biplots illustrate the internal preference mapping relationships between the Kisra products (*blue points*) and assessors (*red points*) for Cluster 3 assessors (24 assessors).

For Cluster 1 (50 assessors), the first two principal component factors (F1 and F2) contributed to 58.02 of the total variation, with F1 explaining 24.01% and F2 accounting for 16.01% (Figure 4A). In cluster 1, Kisra prepared from the Dahab cultivar received the highest overall liking score (~8 “like very much”), followed by Kisra made from the Dabar cultivar and the Dahab + Dabar blend. The Dahab + Arfagadmek blend also received positive preferences, although its score was significantly lower than that of the Dahab alone. Kisra, prepared from Arfagadmek, Wad Ahmed, and Korokolo as standalone cultivars, as well as blends such as Dahab + Korokolo and Dahab + Wad Ahmed, recorded significantly lower preference scores (Figure 4A). In Cluster 2 (26 assessors), the first two principal components (F1 and F2) accounted for 52.04% of the total variation, with F1 explaining 32.42% and F2 contributing 19.61% (Figure 4B). In this cluster, Kisra, made from Dahab, achieved the highest overall liking score, followed by Kisra, made from the Dabar cultivar and the Dahab + Wad Ahmed blend. Meanwhile, Kisra made from Arfa-gadamek recorded the lowest preference score, which was significantly lower than that of all other samples (Figure 4B). The Cluster 3 assessors (24 participants) demonstrated preferences captured by PCA, with F1 and F2 accounting for 54.63% of the total variation (F1 explained 34.85% and F2 explained 19.77%). In cluster 3, Kisra made from Dahab and the Dahab + Korokolo blend received the highest preference scores (~8, “like very much”), significantly exceeding those for Kisra made from Dabar, Korokolo, and Wad Ahmed as standalone cultivars. The Dahab + Wad Ahmed blend had the lowest preference score (“like slightly”) (Figure 4C).

### 3.4 Effects of the demographic profiles on overall liking scores

**Figure 5:**
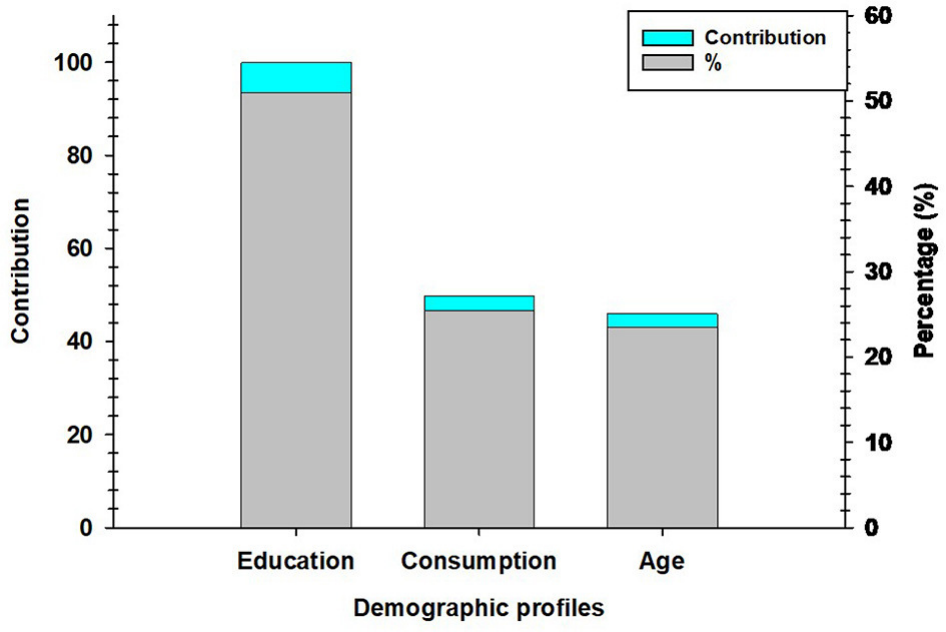
Contribution and percentage of assessors' demographic profile, namely education level, Kisra consumption pattern, and age on overall liking score.

Figure 5 illustrates the contribution and percentage of assessors' demographic profiles, namely education level, consumption pattern, and age, on the overall liking scores for the nine Kisra products. Among the three demographic factors, education level had the highest contribution (100%), accounting for 51% of the total influence. The consumption pattern was second, contributing 50 and 25.5%, respectively. Age had the lowest contribution, with a value of 46.1 and a corresponding percentage of 23.5% (Figure 5).

### 3.5 RATA assay

The results of the three-way ANOVA showed significant variations among the Kisra products for several RATA attributes, including porousness, lightness, darkness, reddishness, sourness, bitterness, and hardness (Table 4). Attributes such as lumpiness, sweetness, thickness, and smoothness did not exhibit significant (Pr > *F* > 0.05) differences among Kisra products (Table 4). With respect to session effects, no significant differences were observed for most RATA attributes except for lumpiness, bitterness, and hardness. Likewise, assessors' impact on RATA attributes was significant for almost all attributes, except lightness (Table 4). The highest porousness (3.48) was observed in Kisra made from Dahab, followed by Wad Ahmed (WA) (3.34), and Dabar (3.26). Kisra, which was made from Korokolo, had the lowest porousness. Kisra, prepared from Dahab, was significantly (Pr > *F* = 0.000) different from those made with Arfa-gadamek, Korokolo, and Dahab + Korokolo blend (Table 5).

**Table 4:** ANOVA summaries for RATA attributes.

		**Porousness**	**Lumpinness**	**Lightness**	**Darkness**	**Reddishness**	**Sweetness**	**Sourness**	**Bitterness**	**Thickness**	**Smoothness**	**Hardness**
*R* ^2^		0.13	0.11	0.14	0.22	0.26	0.21	0.14	0.21	0.14	0.14	0.22
*F*		2.29	1.94	2.55	4.49	5.39	4.18	2.47	4.05	2.58	2.47	4.29
Pr > *F*		< 0.0001	< 0.0001	< 0.0001	< 0.0001	< 0.0001	< 0.0001	< 0.0001	< 0.0001	< 0.0001	< 0.0001	< 0.0001
**Product**	F	4.09	0.79	22.86	44.21	55.86	0.68	7.13	4.41	0.68	1.24	3.98
	Pr > F	< 0.0001	0.608	< 0.0001	< 0.0001	< 0.0001	0.706	< 0.0001	< 0.0001	0.709	0.271	0.000
**Sessions**	F	0.70	19.42	1.45	0.74	0.01	1.93	0.00	16.58	1.84	1.24	27.47
	Pr > F	0.403	< 0.0001	0.229	0.389	0.910	0.165	0.985	< 0.0001	0.175	0.266	< 0.0001
**Assessors**	F	2.16	1.86	0.92	1.32	1.37	4.49	2.12	3.89	2.75	2.59	4.08
	Pr > F	< 0.0001	< 0.0001	0.687	0.022	0.011	< 0.0001	< 0.0001	< 0.0001	< 0.0001	< 0.0001	< 0.0001

Signification codes: 0 < “^***^” < 0.001 < “^**^” < 0.01 < “^*^” < 0.05 < “.” < 0.1 < “ ” < 1.

**Table 5:** Analysis of RATA descriptors of Kisra products.

**RATA terms**	**AG-8**	**Dab**	**Dah**	**Dah + AG-8**	**Dah + Dab**	**Dah + Koro**	**Dah + WA**	**Koro**	**WA**	**Pr > F(Product)**
Porousness	30.01^bc^	30.26^abc^	30.48^a^	3.08^abc^	3.12^abc^	2.96^bc^	3.11^abc^	20.90^c^	30.34^ab^	0.000
Lumpiness	20.66^a^	20.75^a^	20.69^a^	2.82^a^	2.67^a^	2.90^a^	2.81^a^	20.70^a^	20.76^a^	0.636
Lightness	20.52^cd^	30.60^a^	30.61^a^	3.02^b^	2.90^bc^	2.92^bc^	2.60^cd^	20.32^d^	20.80^bc^	< 0.0001
Darkness	30.18^ab^	20.10^d^	10.91^d^	2.57^c^	2.55^c^	3.10^b^	3.47^ab^	30.52^a^	30.28^ab^	< 0.0001
Reddishness	30.16^b^	10.90^d^	10.88^d^	2.44^c^	2.46^c^	2.97^b^	3.33^b^	30.79^a^	30.27^b^	< 0.0001
Sourness	20.66^bc^	20.41^c^	30.25^a^	2.94^ab^	2.86^ab^	2.55^bc^	2.76^bc^	20.77^bc^	20.90^ab^	< 0.0001
Sweetness	30.11	30.13	30.01	2.96	3.06	2.99	3.19	30.04	30.13	0.754
Bitterness	20.13^abc^	10.87^bc^	10.81^c^	2.24^ab^	2.13^abc^	2.04^abc^	2.03^abc^	20.02^a^	20.40^a^	0.000
Thickness	20.61	20.68	20.73	2.75	2.80	2.70	2.80	20.85	20.76	0.724
Smoothness	30.23	30.11	30.21	3.09	3.28	3.15	3.14	20.99	20.99	0.321
Hardness	20.40^abc^	20.37^abc^	20.30^bc^	2.24^c^	2.44^abc^	2.56^abc^	2.68^ab^	20.74^a^	20.61^abc^	0.001

Values followed by different superscript letters in the same row represent significant differences (*P* ≤ 0.05) according to Tukey's test. AG-8, Arfa-gadamek; Dab, Daber; Dah, Dahab; Dah+ AG-8, Dahab and Arfagadamek blend; Dah + Dab, Dahab and Dabar blend; Dah + Wa, Dahab and Wad Ahmed blend; Koro, Korokolo; WA, Wad Ahmed.

Significant differences (Pr > *F* < 0.0001) were observed among the nine Kisra products in terms of lightness. Kisra, made from Dahab and Dabar, had the highest lightness, while Korokolo had the lowest. An inverse trend was observed in the dark condition. Kisra made from Korokolo had the highest score for reddishness, which was significantly higher (Pr > *F* < 0.0001) than other Kisra products. On the other hand, Kisra, made from Arfa-gadamek, Wad Ahmed cultivars, Dahab + Korokolo, and the Dahab + Wad Ahmed blend, showed similar reddishness. Similarly, Kisra from the Dahab + Arfa-gadamek and Dahab + Dabar blends displayed comparable reddishness, whereas the lowest score was recorded for Kisra made from Dahab and Dabar (Table 5). The highest sourness intensity (3.25) was recorded for Kisra prepared from Dahab, whereas the lowest intensity (2.41) was observed for Kisra prepared from Dabar. No significant differences in sourness were found between Kisra made from Dahab, Wad Ahmed, Dahab + Arfa-gadamek, and Dahab + Dabar. Kisra from Dabar, Dahab + Korokolo, and Dahab + Wad Ahmed had similar sourness levels (Table 5).

Kisra made from Wad Ahmed had the highest bitterness intensity (2.40), which was significantly different (< 0.0001) from that of the Dahab, Dabar, Korokolo, and Dahab + Korokolo blends. The lowest bitterness was observed for Kisra, which was obtained for Dahab. Blending Wad Ahmed with Dahab significantly reduced bitterness intensity (Table 5).

The highest hardness intensity (2.74) was recorded in Kisra made from Korokolo, whereas the lowest (2.24) was observed in the Dahab + Arfa Gamdamek blend. Kisra from Korokolo exhibited a significantly (Pr > 0.0001) higher hardness than the Arfa-gadamek, Dabar, Dahab, and Dahab + Arfagademk blends (Table 5).

**Figure 6:**
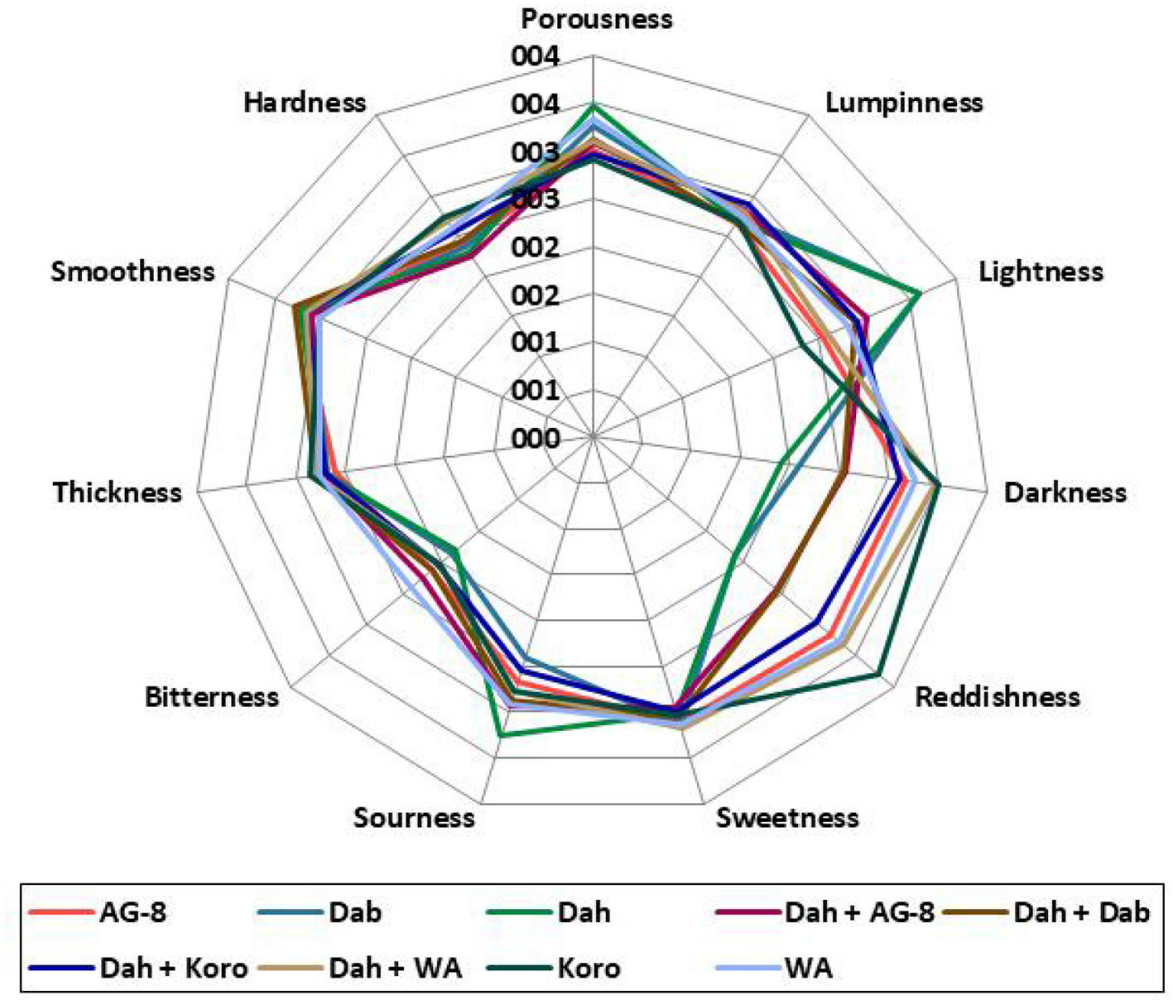
Radar plot with average ratings of the RATA sensory attributes (on a scale from 1 to 5, with 1 = “slightly applicable” to 5 = very applicable) of nine Kisra product samples. For more information on the sorghum cultivar, see Figure.

The sensory characteristics of several Kisra samples, such as hardness, lumpiness, lightness, reddishness, sweetness, sourness, and bitterness, are shown in the radar chart in Figure 6. Dahab exhibited the highest porousness score (3.48), indicating a more open-textured Kisra, whereas the Dahab + Korokolo blend (2.97) and Korokolo blend (2.9) had the lowest scores, suggesting a denser texture. Lumpiness varied slightly among samples, with the Dahab + Arfa-gadamk (2.83) and Dahab + Wad Ahmed (2.82) blends displaying the highest values, indicating a rougher texture (Figure 6). Thickness and hardness were most pronounced in Korokolo (2.83 thickness, 2.75 hardness) and the Dahab + Wad Ahmed blend (2.82), suggesting a firmer consistency. In contrast, Arfa-gadamk (2.61, 2.40 hardness) and Dahab (2.73 thickness, 2.30 hardness) exhibited softer textures (Figure 6). Smoothness was the highest in the Dahab + Dabar and Arfa-gadamk groups, indicating a more refined mouthfeel. Lightness was most notable in Arfa-gadamk and Dahab, whereas Korokolo and Dahab + Wadahmed had the darkest appearances. Reddishness was exceptionally high in Korokolo and Dahab + Wadahmed, indicating a less pronounced red hue. Flavor attributes were relatively consistent across samples, with Dahab + Wadahmed and Dabar scoring highest. The sourness was most noticeable in Dahab, suggesting a tangier profile. Bitterness showed less variation but was highest in Wad Ahmed and Dahab + Arfagadamk (Figure 6).

### 3.6 Correspondence analysis (CA)

**Figure 7:**
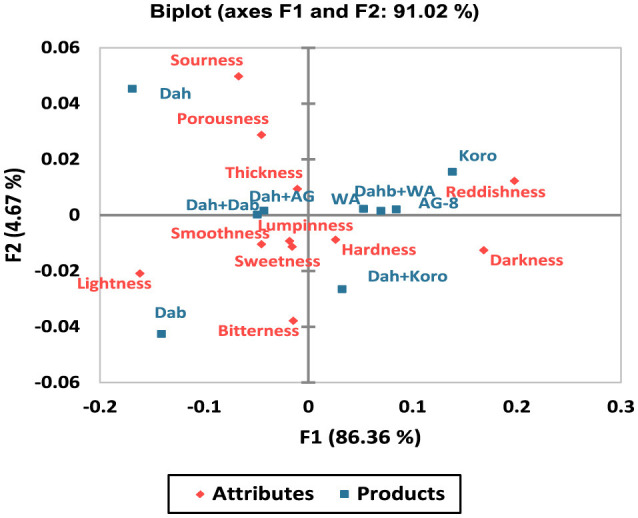
Sensory map obtained through multivariate correspondence analysis using RATA. *AG-8*, Arfa-gadamek; *Dab*, Daber; *Dah*, Dahab; *Dah*+ *AG-8*, Dahab and Arfagadamek blend; *Dah* + *Dab*, Dahab and Dabar blend; *Dah* + *Wa*, Dahab and Wad Ahmed blend; *Koro*, Korokolo; *WA*, Wad Ahmed.

Results from the sensory map, derived from multivariate correspondence analysis using the RATA technique, revealed that the two factors (F1 and F2) accounted for 91 % of the total variation, with F1 contributing 86.36% and F2 explaining 4.67% of the variation (Figure 5). Kisra products prepared from Arfa-gadamek, Korokolo, Wad Ahmed, and the Dahab + Wad Ahmed blend were positioned in the upper right quadrant, closely associated with reddishness. In contrast, Kisra from Dabar was located in the far-left quadrant and was strongly linked to lightness and bitterness. Kisra products made from Dahab and its blends with Dabar and Arfagadmek were situated in the upper-left quadrant, showing strong associations with sourness, porousness, and thickness. The Dahab + Korokolo blend was positioned in the lower right quadrant, reflecting a strong association between hardness and darkness (Figure 7).

### 3.7 Weight of the assessors

**Figure 8:**
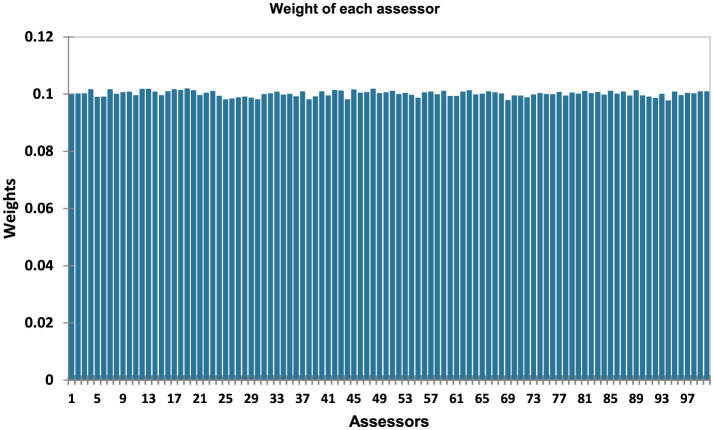
Weight of each assessor participating in RATA test.

The assessor weights were calculated according to the RATA model to determine their contribution to consensus configuration. Higher weights indicate a strong alignment with the overall consensus, whereas lower weights indicate atypical responses. This approach ensures that the results are representative of the majority opinion while identifying outliers, thereby enhancing the robustness and accuracy of RATA interpretation. The RATA model showed an assessor repeatability of 0.994, while the homogeneity and global error values were 0.904 and 9.617, respectively, confirming the reliability and fairness of data interpretation (Table 6). Notably, most assessors had weights ranging between 0.9098 and 0.102, with the majority clustered around 0.100, indicating balanced distribution and consistency across participants (Figure 8).

**Table 6:** Assessors repeatability, homogeneity/panel consistency test and the global error of the RATA model.

**Item**	**Value**	***p*-value**
Assessors' repeatability	0.99	^***^
Homogeneity/panel consistency test/	0.90	^***^
Global error	9.62	

Signification codes: 0 < “^***^” < 0.001.

## 4 Discussion

This study evaluated nine Kisra products prepared from five sorghum cultivars, including the biofortified cultivar Dahab and its blends, using sensory feedback from 100 untrained assessors from diverse socioeconomic backgrounds. Based on a preliminary focus group discussion, 11 sensory attributes were incorporated into the RATA method to thoroughly understand Kisra's sensory characteristics. To our knowledge, this is the first report to employ a rapid sensory tool such as RATA for evaluating Kisra, a staple traditional fermented flatbread widely consumed across Sudan, particularly in rural communities where micronutrient deficiencies are prevalent. Incorporating biofortified sorghum, such as the recently released Dahab cultivar, into the Kisra preparation supplies a culturally acceptable and innovative solution to combat hidden hunger. This approach aligns with consumer preferences and effectively addresses micronutrient deficiencies while preserving the traditional and cultural significance of Kisra as a staple food.

Our study is the first to demonstrate the effectiveness of the RATA approach in evaluating traditional and fermented sorghum-based foods, highlighting its applicability in resource-limited settings. The results showed that untrained consumers could effectively discriminate between the nine Kisra product samples, reinforcing the feasibility of RATA as an alternative to traditional sensory evaluation methods. These findings align with those of previous studies, such as Dinnella et al. (36), which suggested that remote sensory testing, when conducted under strict sensory protocols, can be a viable substitute for laboratory-based evaluations for trained assessors and naïve consumers. Similarly, Rabitti et al. (20) reported that the RATA method does not require trained sensory panels, as it effectively captures consumer perceptions even among untrained participants. Additionally, Ares et al. (37) emphasized that RATA can yield insights comparable to traditional descriptive analysis (DA), depending on the sensory complexity of the product. RATA, conducted with naïve consumers for more straightforward sensory profiles, provides results similar to those obtained through classical DA.

Our study demonstrated a high repeatability index (0.994), strong homogeneity (0.904), and minimal global error (9.617), validating the reliability and accuracy of the RATA method in effectively capturing feedback from untrained or inexperienced assessors during the evaluation of nine Kisra products. The scores of most assessors ranged from 0.098 to 0.0102, indicating that the RATA method was objectively designed and free of preferential bias, thereby enhancing the credibility of the results. These findings align with those of Giacalone and Hedelund (22), who reported RATA's high reproducibility and validity as a sensory profiling tool even with semi-trained assessors. They emphasized that RATA's inherent characteristics are advantageous in industrial contexts, where small, semi-trained panels are employed and sensory evaluations are constrained by time and limitations. Kisra made from the biofortified sorghum cultivar Dahab consistently received the highest overall liking score (~8, “like very much”), significantly outperforming the other Kisra samples. These findings demonstrate the potential of biofortified sorghum to prevent malnutrition while maintaining consumer preference. The acceptability of Dahab in Kisra preparation aligns with the studies by Birol et al. (12) and Talsma et al. (14), who reported higher consumer acceptability of biofortified foods in low- and middle-income countries. The rapid sensory evaluation methods employed in this study can further aid plant breeders in developing biofortified varieties tailored to meet consumer acceptance and expectations, thereby increasing the likelihood of adoption (38).

Blends such as Dahab + Dabar and Dahab + Arfa-gadamek and Kisra products received overall liking scores (~7, “like moderately”), indicating their potential for scaling as culturally acceptable and nutritionally beneficial food products, which could combat micronutrient deficiencies and provide local consumers with familiar tastes and culturally preferred sorghum-based foods. This finding aligns with a report by Makawi et al. (39), which indicated a significant reduction in antinutritional factors and improved sensory attributes of Kisra prepared using a blend of fermented sorghum and baobab fruit pulp flour. Similarly, Mariod et al. (40) demonstrated that incorporating *Monechma ciliatum* seeds as a food supplement with sorghum flour in a 1:10 ratio significantly enhanced the nutritional and sensory qualities of Kisra products.

Inconsistencies in preference segmentation can lead to misidentification of market opportunities and improper product optimization, contributing to a 75–80% failure rate of new products in the market (46, 47). This study employed agglomerative hierarchical cluster analysis to segment assessors based on their overall hedonic scores for the nine Kisra products. Grouping assessors into three distinct clusters enhanced the precision of sensory evaluation, provided valuable insights into preference patterns, and supported the optimization of biofortified Kisra formulations tailored to specific consumer segments.

Gere (47) emphasized the importance of selecting appropriate clustering algorithms, determining optimal cluster numbers, and validating approaches to ensure reliable consumer preference predictions. Our study revealed that educational level was the most influential demographic factor in shaping overall liking scores, contributing 51% of the total variance, followed by consumption frequency (25.5%) and age (23.5%).

The education segmentation analysis, based on Table 1, indicates that higher education levels correlate with more refined sensory perceptions and specific product preferences. Assessors who attended intermediate school or higher demonstrated greater sensitivity to sensory attributes, which influenced their hedonic ratings. This trend aligns with the findings of Helmefalk and Eklund (41), who reported that individuals with higher educational levels tend to have more developed preferences for sensory attributes, impacting their overall evaluation of food products.

The variation in sensory perception due to education can be attributed to greater exposure to diverse food textures, flavors, and preparation methods, which influence the discriminatory ability in sensory evaluation. Lower-educated or illiterate assessors, who comprised 41% of our panel, exhibited more generalized preferences, possibly due to limited exposure to alternative sorghum-based Kisra formulations. This highlights the importance of consumer education in promoting acceptance of biofortified products, particularly in rural communities.

Age was the third most significant demographic factor influencing the overall liking scores, contributing 23.5% of the variance. Contrary to the findings of other sensory studies (42, 43), where elderly consumers (61+ years) gave higher liking scores, our study found no significant increase in overall liking among older assessors. One possible explanation for this deviation is that the elderly consumers in our study had a strong preference for traditional Kisra formulations, making them less receptive to novel biofortified sorghum-based variations. Unlike fruit cakes and orange juice, where higher sugar content and familiarity positively influenced elderly preference, biofortified Kisra may have introduced subtle differences in texture, sourness, or mouthfeel that required an adaptation period before being widely accepted. This underscores the need for targeted sensory education and gradual product integration strategies to enhance acceptance of biofortified sorghum products among older populations.

Consumption habits significantly shaped the overall liking scores, accounting for 25.5% of the variance. Assessors who consumed Kisra daily or multiple times per week exhibited higher acceptance levels, likely due to greater familiarity with the sensory attributes of sorghum-based foods. This aligns with Michon et al. (43), who found that frequent consumption of jam-filled cakes resulted in higher liking scores, as consumers with habitual exposure to specific flavors and textures developed a stronger affinity for those products. Similarly, Ikegaya et al. (44) emphasized the importance of considering demographic factors when interpreting sensory data, as repeated exposure can positively influence consumer preferences. In contrast, assessors who reported consuming Kisra only once per week or less frequently showed greater variability in their hedonic ratings, possibly because of lower familiarity with the sensory attributes of the different sorghum cultivars. This finding highlights the importance of increasing consumer exposure to biofortified Kisra products to ensure that gradual integration into traditional diets enhances acceptability.

In this study, the sensory map obtained from the multivariate correspondence analysis (CA) using the RATA model accounted for 91% of the total variation, demonstrating the effectiveness of the model in evaluating consumer perceptions of kisra products. This high total variation score reflects the comprehensive nature of the RATA format, which provides a nuanced understanding of assessor preferences essential for product development and marketing strategies (35). Similarly, Sales et al. (45) indicated the effectiveness of combining RATA with CA and ANOVA to generate sensory maps for kombucha products.

The CA results indicated that Kisra made from the biofortified Dahab cultivar and its blend Dabar + Arfa-gadamek exhibited strong positive associations with sensory attributes such as sourness, porousness, and thickness, positioned in the upper-left quadrant. These findings suggest that biofortified sorghum, particularly Dahab, can be optimized to align better with consumer tastes and preferences.

The sensory map revealed the distinct attributes of the nine Kisra products across the four quadrants. For instance, Kisra made from the Sudanese Feterita sorghum cultivars, namely Arfa-gadamek, Korokolo, and Wad Ahmed, and the Dahab + Wad Ahmed blend positioned in the upper-right quadrant, strongly linked to reddishness, appealing to assessors with a preference for color attributes. The reddish hue is typically attributed to the presence of pigmented tannin-condensed testa in Feterita sorghum grains (23), which are widely preferred by many Sudanese because of their high nutritional value and associated health benefits (24). Leveraging these attributes while balancing sensory preferences and dietary goals could expand the appeal of biofortified kisra across diverse consumer segments.

## 5 Conclusion

This study indicates the effectiveness of the RATA method in evaluating sensory preferences and understanding consumer perceptions of Kisra products. These findings demonstrate that the biofortified Dahab sorghum cultivar has strong potential for addressing malnutrition in rural communities while catering to diverse consumer preferences. Hierarchical clustering analysis based on Euclidean distance grouped assessors into three distinct clusters with varying preferences, providing valuable product development and commercialization insights. Among the demographic factors, educational level had the strongest influence on overall liking, followed by consumption pattern and age, indicating the need for tailored marketing and awareness strategies when promoting biofortified kisra. The combination of hedonic scores with the RATA method revealed that Dahab-based Kisra was strongly associated with desirable sensory attributes, including porousness, thickness, and sourness, reinforcing its suitability for widespread adoption. This study recognizes that additional research must be conducted regarding these promising outcomes. Future research should include well-trained sensory panels to verify the accuracy of the RATA results obtained by untrained assessors. Evaluating consumer acceptance through time-based studies would improve the knowledge of stable preference patterns. Additional nutritional and biochemical studies on Kisra products using advanced omics techniques would support the sustainability of sorghum as a micronutrient deficiency prevention method.

## Data Availability

The raw data supporting the conclusions of this article will be made available by the authors, without undue reservation.
